# Support for research towards understanding the population health vulnerabilities to vector-borne diseases: increasing resilience under climate change conditions in Africa

**DOI:** 10.1186/s40249-017-0378-z

**Published:** 2017-12-12

**Authors:** Bernadette Ramirez, on behalf of the TDR-IDRC Research Initiative on Vector Borne Diseases and Climate Change, Yeya Toure, Johannes Sommerfeld, Florence Fouque, Thierry Baldet, Zee Leung, Dominique Charron, Diarmid Campbell-Lendrum, Magaran Bagayoko, Madeleine Thomson, Pietro Ceccato, Moses Chimbari, Benson Estambale, Paul Gwakisa, John Hargrove, Brama Kone, Mario Henry-Rodriguez, Moses Bockarie

**Affiliations:** Vectors, Environment and Society Unit, Special Programme for Research and Training in Tropical Diseases (TDR), World Health Organization (WHO), Geneva, Switzerland

**Keywords:** Vector-borne diseases, Climate change, Adaptation, Resilience, Malaria, Schistosomiasis, Rift Valley fever, Human African trypanosomiasis

## Abstract

**Background:**

Diseases transmitted to humans by vectors account for 17% of all infectious diseases and remain significant public health problems. Through the years, great strides have been taken towards combatting vector-borne diseases (VBDs), most notably through large scale and coordinated control programmes, which have contributed to the decline of the global mortality attributed to VBDs. However, with environmental changes, including climate change, the impact on VBDs is anticipated to be significant, in terms of VBD-related hazards, vulnerabilities and exposure. While there is growing awareness on the vulnerability of the African continent to VBDs in the context of climate change, there is still a paucity of research being undertaken in this area, and impeding the formulation of evidence-based health policy change.

**Main body:**

One way in which the gap in knowledge and evidence can be filled is for donor institutions to support research in this area. The collaboration between the WHO Special Programme for Research and Training in Tropical Diseases (TDR) and the International Centre for Research and Development (IDRC) builds on more than 10 years of partnership in research capacity-building in the field of tropical diseases. From this partnership was born yet another research initiative on VBDs and the impact of climate change in the Sahel and sub-Saharan Africa. This paper lists the projects supported under this research initiative and provides a brief on some of the policy and good practice recommendations emerging from the ongoing implementation of the research projects.

**Conclusion:**

Data generated from the research initiative are expected to be uptaken by stakeholders (including communities, policy makers, public health practitioners and other relevant partners) to contribute to a better understanding of the impacts of social, environmental and climate change on VBDs(i.e. the nature of the hazard, vulnerabilities, exposure), and improve the ability of African countries to adapt to and reduce the effects of these changes in ways that benefit their most vulnerable populations.

**Electronic supplementary material:**

The online version of this article (10.1186/s40249-017-0378-z) contains supplementary material, which is available to authorized users.

## Multilingual abstracts

Please see Additional file 1 for translations of the abstract into the five official working languages of the United Nations.

## Background

Diseases transmitted to humans by vectors (mosquitoes, flies, sandflies, triatomine bugs, ticks, fleas, snails, etc.) such as malaria, dengue, yellow fever, chikungunya, Zika, West Nile virus, lymphatic filariasis, leishmaniasis, Chagas disease, Crimean-Congo haemorrhagic fever, tick-borne encephalitis, typhus, Lyme disease, human African trypanosomiasis, onchocerciasis and schistosomiasis account for 17% of all infectious diseases and remain significant public health problems [1]. More than half of the world’s population are at risk of vector-borne diseases (VBDs); each year, more than a billion people are infected and 1 million people die from these diseases [1, 2]. Aside from deaths, many VBDs also cause considerable debilitation and suffering of affected populations. Through the years, great strides have been recorded towards combatting VBDs, most notably through large scale and coordinated control programmes, which have contributed to the decline of the global mortality attributed to VBDs. However, the health- and socio-economic impacts of VBDs, especially among vulnerable populations that are already facing the challenges of extensive poverty, fragile ecosystems, high population growth and non-existent, sub-optimal or failing health and social security systems, continue to be of grave concern.

The geographic distribution of VBDs is influenced by a complex dynamic of environmental and social factors and their changing impact on the transmission and burden of VBDs through effects on their vectors, intermediate hosts and reservoirs. Current changes in the long-term trends of regional weather patterns, referred to as ‘climate change’, are adding new pressures to VBDs [3]. The impacts are anticipated to be significant, in terms of VBDs-related hazards, vulnerabilities and exposure. There is existing evidence to suggest that climate change impacts will substantially increase burdens on those people that are already vulnerable to climate extremes, such as those in the African continent [4], notably the drylands in the sub-Saharan region. Already this region is bearing the brunt of the disease burden due to VBDs and yet this region is also challenged by environmental changes, including climate change [5], which are perceived to induce multiple threats to development in the African continent. With climate change adding new pressure on already fragile ecosystems and populations, the African continent may face increased threats from VBDs since important disease vectors/intermediate hosts (e.g. insects, snails) depend on water and other environmental factors. Increasingly scarce water sources concentrate people and animals in specific locations, which can become hotspots for VBD transmission.

There are several broad policy frameworks that call for action to address the impact of climate change on health. One such framework is the Paris Agreement for climate actions and building resilience, which is one of the essential facets contributing to the achievement of the United Nations Sustainable Development Goals 2016–2030 [6]. Likewise, there are several global mechanisms such as the WMO (World Meteorological Organization) Global Framework of Climate Services (GFCS) for coordinated actions for the enhancement of the quality, quantity and application of climate services; the UNFCC (United National Framework – Convention on Climate Change), the International Health Regulations for the improvement of the capacity of countries to respond to public health threats; and the Climate for Development in Africa Initiative (ClimDev-Africa) for overcoming climate information gaps, for analyses leading to adequate policies and decision-making. In Africa, emphasis on the co-benefits of responding to health threats in the context of a changing environment is highlighted in the Libreville Declaration of 2008, followed by the Luanda Commitment of 2010. Signed by African ministers responsible for health and environment, these two documents acknowledged the serious threat to health of environmental risks (including climate change) and the urgency for multisectoral actions and linkages in the health and environment sectors through the Framework and Plan of Action for Public Health Adaptation to Climate Change in the African Region [7].

Although these policy frameworks are available, the lack of knowledge and evidence on the possible impacts of climate change on VBDs in Africa remains a serious obstacle to evidence-based health policy change [8–13]. The growing awareness of the African region’s vulnerability to VBDs in the context of climate change and the paucity of research being undertaken in this area [12] have given impetus for the WHO Special Programme for Research and Training in Tropical Diseases (TDR) and the International Centre for Research and Development (IDRC) to partner in support of a new portfolio of research projects in the Sahel and Sub-Saharan Africa through the TDR IDRC research initiative on VBDs and Climate Change [13]. Additional technical collaboration is provided by WHO’s Department of Public Health and Environment, WHO’s Regional Office for Africa and the International Research Institute for Climate and Society.

Building on over 10 years of collaboration, TDR and IDRC had established this research initiative to address VBDs in the broader development context of human vulnerability to climate change. This research initiative is expected to result in knowledge, research capacity, collaboration and policy advice products that can be used throughout Africa and other regions.

### Main text

Five projects comprise the TDR IDRC Research Initiative (see Table 1 for a list of projects). The projects, led by researchers from research institutions in Botswana, Cote d’Ivoire, Kenya, Mauritania, South Africa, Tanzania and Zimbabwe, and in collaboration with existing disease control programmes in the context of the National Adaptation Plans for Climate Change (NAPs) in Africa [14], were focused on addressing malaria, schistosomiasis, human African trypanosomiasis and Rift Valley fever. The projects provided a holistic research perspective to elucidate how environmental and socio-economic change affects transmission dynamics and disease burden of VBDs through changes in vector ecology, human ecology, social organization, demography and health systems. Vulnerability and resilience of specified socio-ecological systems (SES) were assessed based on multi- and transdisciplinary models and frameworks. In addition, social and health systems research were expected to illuminate and analyse community structures, dynamics and resilience, health systems performance and response to VBDs in the broader multisectoral stakeholder context.

**Table 1:** List of Research Projects

Project title	Vector-borne disease/s (and study sites)	Principal Investigator
Social, environmental and climate change impact of vector-borne diseases in arid areas of Southern Africa Some publications from this research project - [5, 9, 15–34]	Malaria and schistosomiasis	*Moses Chimbari*, College of Health Sciences, University of Kwazulu-Natal, Durban, South Africa
	• Botswana (3 villages in Ngarange and Shakawe) • South Africa (uMkhanyakude: Mgedula, Ndumo and Makanis villages) • Zimbabwe (Gwanda district: Makwe and Byuma villages)	
Early warning systems for improved human health and resilience to climate sensitive vector-borne diseases in Kenya Some publications from this research project -[35–37].	Malaria and Rift Valley fever	*Benson Estambale*, Jaramogi Oginga Odinga University of Science and Technology, Bondo, Kenya
	• Kenya (Kabarnet town in Baringo County	
Predicting vulnerability and improving resilience of the Maasai communities to vector-borne infections: an ecohealth approach in the Maasai Steppe ecosystem Some publications from this research project -[38, 39].	African trypanosomiasis	*Paul Gwakisa,* Nelson Mandela African Institute of Science and Technology (NMAIST), Arusha, Tanzania and The Genome Science Centre and Department of Veterinary Microbiology and Parasitology, Faculty of Veterinary Medicine, Sokoine University of Agriculture, Morogoro, Tanzania
	• Tanzania (Oltukai village located between Manyara Ranch and Lake Manyara National Park in Monduli district; Emboreet, Loiborsiret and Kimotorok villages bordering Tarangire National Park in Simanjiro district)	
Human African trypanosomiasis: alleviating the effects of climate change through understanding the human-vector-parasite interactions Some publications from this research project-[40–53]	African trypanosomiasis	*John Hargrove,* South African Centre of Excellence in Epidemiological Modelling and Analysis (SACEMA), University of Stellenbosch, South Africa
	• Zimbabwe (Rekomitjie Research Station located in Mana Pools National Park, Zambezi Valley, Mashonaland West Province; and Vuti village located on the fringes of the wildlife areas) • Tanzania (Villages in the Ikorongo-Grumeti area to the west of the Serengeti National Park)	
Vulnerability and resilience to malaria and schistosomiasis in northern and southern fringes of the Sahelian belt in the context of climate change Some publications from this research project - [54–56]	Malaria and schistosomiasis	*Brama Kone,* Centre Suisse de Recherches Scientifiques en Côte d’Ivoire, Abidjan, Côte d’Ivoire
	• Cote d’Ivoire (Korhogo) • Mauritania (Kaedi)	

Implementation of the individual research projects is still ongoing through these three phases: Phase 1 - description and characterization of the complex socio-ecological system in African dryland water systems, not only for their environmental, social and climate change aspects but also VBD transmission dynamics and disease burden; Phase 2 - investigation of the changing context of the environmental, social, economic and climate change conditions and its impact on VBD transmission and burden; Phase 3 - development of community-based adaptation strategies and decision-support processes and tools for reducing population health vulnerabilities to VBDs. For the interested reader, a few emerging publications from the projects are listed on Table 1.

Capacity building is central to this research initiative. A Special Project Team was established to provide scientific and technical guidance to the research teams for the implementation of their research projects. Capacity building efforts (for research, ethics and gender-based data analysis), targeting research teams, communities, public health practitioners and those from the environment sector, were advanced through workshops and training courses. A web-based platform, vbd-environment.org, was specifically developed for knowledge-sharing and for dissemination of information..Other training opportunities were also available to support students at the Masters, PhD and post-doctoral levels (59 students total; 56% men and 44% women).

Research uptake is key in facilitating the use of research evidence by stakeholders which include communities, policy makers, practitioners and other relevant partners. Early and continuous engagement with stakeholders was at the core of the projects’ implementation process. A Research Uptake Meeting held last April 2017, in Brazzaville, Republic of Congo at the WHO Regional Office for Africa, was one of several notable occasions that provided a venue to engage research teams and their respective sectoral partners (from the ministries of health and environment) to discuss draft policy briefs based on emerging evidence and knowledge from the research projects. This meeting laid the groundwork for future continued engagement that is expected to promote a comprehensive, integrated and multisectoral approach to increasing resilience to VBDs under climate change conditions. During this meeting, the ministry officials from the health and environment sectors had acknowledged the usefulness of research towards contributing to further improvement of the implementation of VBD control programmes and for informing/updating the national health and environment strategic plans.

The following provides a brief on some of the policy and good practice recommendations emerging from the ongoing implementation of the research projects:

### Social, environmental and climate change impact of vector-borne diseases in arid areas of southern Africa

This project, undertaken in specific vulnerable communities in arid areas of Botswana, South Africa and Zimbabwe, had sought to determine the impacts of socio-economic, environmental, climatic, bionomic and institutional factors on malaria and schistosomiasis. This project had demonstrated, through a community engagement framework (involving active participation of community members together with researchers and public health practitioners) [17, 33, 57–59], an effective community-based early warning system for malaria and schistosomiasis, which incorporates observations on the changing climate (droughts, floods, heat waves and rainfall variability). This project was also able to apply Geographic Information System (GIS) and remote sensing tools, used at micro-geographic scale, to locally identify local hotspots for schistosomiasis transmission [16, 18, 34, 37].

### Early warning systems for improved human health and resilience to climate sensitive vector-borne diseases in Kenya

The goal of this project was to develop a framework for an integrated, community-based early warning system for improved human health and resilience to climate–sensitive VBDs (malaria and Rift Valley fever) in Kenya. This project had contributed to the development of spatial distribution maps for the prediction of disease risk in affected communities [21, 24].

This project had shown that, while there is good knowledge of malaria in the community, the confounding myths and misconceptions may adversely influence treatment-seeking behaviour and effective control [36]. Non-adherence to malaria medication may also lead to drug resistance and other adverse side effects. Given the just confirmed perennial transmission of malaria and a high rate of asymptomatic cases in the riverine zone, there is potential for the emergence of transmission hot spots. There is thus a need for routine surveillance, appropriate management of the disease, particularly among primary school children, and expansion of malaria control initiatives into emerging hot spots such as the riverine zone. Mosquito net coverage is not adequate and the utilization is not optimal and can be improved. Populations living in mud-walled grass-thatched houses with open eaves are at highest risk of malaria infections in indoor environments. Finally, where microscopic examination is unavailable, Rapid Diagnostic Tests (RDTs) provide alternative cost effective, accurate and rapid detection of malaria.

This project had shown that climate change can have an impact on the spatial distribution of RVF vectors by expanding their realized niche, thus putting more populations at risk [39]. These findings can be used by policy makers, government agencies, medical and veterinary personnel in planning the targeted prevention and management of RVF.

### Predicting vulnerability and improving resilience of the Maasai communities to vector-borne infections: an ecohealth approach in the Maasai steppe ecosystem

The goal of this project was to use climate and land use models to predict potential hotspots of infection with African trypanosomiasis. This project had shown that African trypanosomiasis remains as a disease of public health importance affecting animals and humans in the Maasai steppe in Tanzania. In this project, it was observed that majority of pastoralists are aware of the animal form of trypanosomiasis; however, knowledge of Human African trypanosomiasis is poor. The changing climate is a major driver of the abundance of tsetse flies and their infection rates. Habitats, vegetation and host availability are strong predictors of tsetse abundance infection rates [38, 40]**.** The presence of reservoirs, anthropo-zoonotic feeding behavior of tsetse flies and changing climate can potentially result in the resurgence of human African trypanosomiasis. It is therefore important to establish monitoring and surveillance, especially within the human-animal interface areas of the Maasai steppe.

### Human African trypanosomiasis: alleviating the effects of climate change through understanding human-vector-parasite interactions

There is a scarcity of detailed biological data that can be used to assess the dependence of the vector and the disease on climatic parameters and how changes in climatic parameters are liable to affect the disease situation. This research project used the unique archives of data and augmented this by additional data from field investigations of man-fly and man-disease contact in Zimbabwe and Tanzania, to understand the spatio-temporal variability of disease threat and how this would likely change at different locations and altitudes in the context of climate change. This project had shown that farmers in western Tanzania are unfamiliar with the transmission of trypanosomiasis and can thus benefit from training, especially on the use of Restricted Application of Pyrethroid (RAP) insecticides as a cost-effective control method [39, 41]. Tsetse populations were observed to be sensitive to climatological changes and therefore, it is imperative that climate variables be monitored together with monitoring of vector population levels and the presence of trypanosomes in the tsetse vectors and mammalian hosts [42–45, 50, 52].

### Vulnerability and resilience to malaria and schistosomiasis in northern and southern fringes of the Sahelian belt (Cote d’Ivorie and Mauritania)

Using the ecohealth approach, this research project aimed to develop, through a community participatory approach, tools and appropriate coping strategies to alleviate the effects of climate change on the transmission of malaria and schistosomiasis. This project had shown that education, social support and governance have a strong impact in building community resilience to VBDs. There was a clear association between rainfall and temperature with the transmission of malaria and schistosomiasis which can inform planning and prioritization of resource allocation for a more effective implementation of VBD control programmes [46, 49, 51].

## Conclusion

VBDs continue to contribute significantly to the global burden of disease, and cause epidemics that disrupt health security and cause wider socioeconomic impacts around the world. All are sensitive in different ways to weather and climate conditions, so that the ongoing trends of increasing temperature and more variable weather threaten to undermine recent global progress against these diseases. Here, we have presented the TDR-IDRC Research Initiative on VBDs and Climate Change as an example of a research portfolio that can contribute to a broader, transdisciplinary, holistic systems-approach to research [57–59] leading to a better understanding of the population health vulnerabilities to vector-borne diseases and towards increasing resilience under climate change conditions in Africa. It is expected that the TDR-IDRC research portfolio will add new knowledge and evidence on VBD impacts under climate change conditions on vulnerable populations, which, in turn may inform the development of practical frameworks, processes and tools for policy-decision making for better risk management and for building on existing VBD control strategies to come up with innovative approaches. This portfolio also is also expected to contribute to building African capacity for transdisciplinary policy-oriented research. The transdisciplinary approach (from conception to organization and to implementation of research projects) is expected to provide an enabling milieu for a process of mutual learning and exchange that involves shareholders and important societal actors (e.g. relevant decision-makers, affected local communities) to transform health concerns into finding solutions together [60–62].

The more pressing need at present is to ensure that VBD control efforts that integrate management of the risks brought by climate change are further strengthened. Political support and financial investments are in order for the re-design of national healthcare system components (human resources, facilities, technology, health information systems and health policies) [63]to enable scaling up and access of innovative preventive approaches to VBD control that combines a comprehensive and coordinated management of climate risks [64]. There is also a need to adopt better mechanisms that will facilitate collaborations between ministries that are responsible for health and environment while empowering public health practitioners, researchers and communities to collaborate in shaping integrated government-wide strategies [65–70].

## Additional file

Additional file 1: Multilingual abstracts in the five official working languages of the United Nations. (PDF 772 kb)
