# Supplementary material for: Support for research towards understanding the population health vulnerabilities to vector-borne diseases: increasing resilience under climate change conditions in Africa
Source: Infect Dis Poverty. 2017 Dec 12;6:164. doi: 10.1186/s40249-017-0378-z (PMC5725740; doi:10.1186/s40249-017-0378-z)
Supplement: Additional file 1: — Multilingual abstracts in the five official working languages of the United Nations. (PDF 772 kb) [file 40249_2017_378_MOESM1_ESM.pdf]

Translation of the abstract into the five official working languages of the United Nations

تقديم الدعم للبحوث المتعلقة بفهم مواطن الضعف الصحية للسكان تجاه الأمراض التي تنتقل بالنواقل: زيادة القدرة على الصمود في ظل ظروف تغير المناخ في أفريقيا

برناديت راميريز

ملخص

خلفية الموضوع: تبلغ نسبة الأمراض المنقولة إلى البشر بواسطة الحشرات 17 بالمئة من مجموع الأمراض المعدية، وهي تشكل مشكلة حقيقية للصحة العامة، على مر السنين، تم اتخاذ خطوات كبيرة نحو مكافحة الأمراض المنقولة بالنواقل عن طريق برامج واسعة النطاق ومنسقة، مما ساهم في انخفاض معدل الوفيات العالمي المتعلق بالأمراض المنقولة بالنواقل، لكن مع التغيرات البيئية الحاصلة، بما فيها تغير المناخ، يتوقع أن يكون تأثير الأمراض المنقولة بالنواقل كبير من حيث المخاطر المتعلقة بهذه الأمراض ومواطن الضعف تجاهها ونسبة التعرض لها، على الرغم من ازدياد الوعي بضعف القارة الأفريقية تجاه هذه الأمراض في ظل تغير المناخ، لا يزال هناك شح في البحوث المتعلقة بهذا المجال مما يعيق صياغة سياسة التغيير الصحية المبنية على الأدلة.

المحتوى: إحدى الطرق التي يمكنها أن تسد النقص المتعلق بالمعرفة وجمع الأدلة هو أن تقوم المؤسسات المانحة بدعم البحوث في هذا المجال، ويستند التعاون بين البرنامج الخاص للبحوث والتدريب في مجال أمراض المناطق الاستوائية (TDR) لمنظمة الصحة العالمية والمركز الدولي للبحوث والتنمية (IDRC) على شراكة دامت أكثر من 10 سنوات في بناء القدرات البحثية في مجال الأمراض الاستوائية، ومن هذه الشراكة تولدت مبادرة بحثية أخرى حول الأمراض المنقولة بالنواقل وتأثير تغير المناخ في منطقة الساحل وجنوب الصحراء الأفريقية، وتحتوي هذه الورقة على قائمة بالمشاريع المدعومة ضمن هذة المبادرة البحثية وموجزاً عن بعض التوصيات المتعلقة بالسياسات والممارسات الجيدة التوصيات الناشئة عن تنفيذ المشاريع البحثية الجارية.

الاستنتاج:

من المتوقع أن تفيد المعطيات التي تم استنتاجها من المبادرة البحثية أصحاب المصلحة (بما في ذلك المجتمعات وصانعي القرار، والممارسين في مجال الصحة العامة والشركاء الآخرين ذوي الصلة)، وأن تسهم في فهم أفضل لتأثير التغييرات الاجتماعية والبيئة والمناخية تغير على الأمراض المنقولة بالنواقل (أي طبيعة المخاطر ومواطن الضعف والتعرض)، وتحسين قدرة البلدان الأفريقية على التكيف مع هذه التغييرات والحد من آثارها بطرق تعود بالنفع على السكان الأكثر عرضه لها.

Translated from English version into Arabic by Madawi Alajmi, through

TRANSLATORS
WITHOUT BORDERS

关于了解媒介传播疾病的人群健康易感性的支持性研究：在非洲气候变化条件下增强抵御力

Bernadette Ramirez

摘要

引言：通过媒介传染给人类的疾病占所有传染病的 17%，仍然是重要的公共卫生问题。多年来，媒介传播疾病（VBD）防治工作已取得了巨大的进步，最主要的是通过大规模的联动控制方案，从而降低了 VBDs 对全球死亡率的贡献。然而，随着环境的变化，包括气候变化的影响，就 VBD 相关危害、脆弱性和暴露方面而言，预计对 VBD 的冲击会比较明显。虽然越来越多的人意识到在气候变化的背景下非洲大陆的脆弱性，但仍然缺乏这方面的相关研究，并阻碍循证卫生政策的改变。

主要内容：填补知识和证据差距的一个方法是捐助机构支持这方面的研究。世界卫生组织热带病研究和培训特别规划署（TDR）与国际研究发展中心（IDRC）的合作是建立在超过 10 年的关于热带疾病领域研究能力建设伙伴关系基础之上的。从这个合作诞生了另一个关于 VBD 的研究计划，即在萨赫勒和撒哈拉以南非洲地区研究气候变化对 VBD 的影响。本文列出了本研究倡议支持下的项目，简要介绍了正在实施的研究项目中所提出的一些政策和良好实践的建议。
结论：研究计划所产生的数据将给利益相关者提供参考（包括社区、政策制定者、公共卫生工作者和其他相关合作伙伴），有助于更好地了解社会、环境和气候变化对 VBD 的影响（即自然界的危害、脆弱性和暴露），提高非洲国家适应和减少这些变化影响的能力，使其最易感人群受益。

Translated from English version into Chinese by Zhou Guan, edited by Pin Yang

CHINESE CENTER FOR DISEASE CONTROL AND PREVENTION
NATIONAL INSTITUTE OF PARASITIC DISEASES

Soutien à la recherche axée sur la compréhension des vulnérabilités en matière de santé publique face aux maladies à transmission vectorielle : accroissement de la résilience dans des conditions de changement climatique en Afrique

Bernadette Ramirez

Résumé

Rappel des faits: Les maladies transmises aux humains par des vecteurs représentent 17 % de toutes les maladies infectieuses et demeurent des problèmes de santé publique importants. Au fil des années, des progrès considérables ont été accomplis pour lutter contre les maladies à transmission vectorielle (MTV), principalement par l'intermédiaire de programmes de contrôle coordonnés et à grande échelle, qui ont contribué au déclin de la mortalité mondiale attribuée aux MTV. Cependant, avec les changements environnementaux, notamment le changement climatique, l'impact sur les MTV devrait être important, en termes de vulnérabilité, d'exposition et de risques liés aux MTV. Alors qu'il existe une prise de conscience croissante de la vulnérabilité du continent africain face aux MTV dans le contexte du changement climatique, il y a toujours un manque de recherches menées dans ce domaine, ce qui entrave la formulation d'un changement de politique de santé fondée sur l'expérience concrète.
Partie centrale: Un des moyens de combler les lacunes en termes de connaissances et de preuves serait, pour les institutions donatrices, de soutenir la recherche dans ce domaine. La collaboration entre le Programme spécial de recherche et de formation concernant les maladies tropicales (TDR) de l'OMS et le Centre de recherches pour le développement international (CRDI) s'appuie sur plus de 10 ans de partenariat dans le renforcement des capacités de recherche dans le domaine des maladies tropicales. De ce partenariat est née une nouvelle initiative de recherche sur les MTV et l'impact du changement climatique au Sahel et en Afrique subsaharienne. Cet article répertorie les projets soutenus dans le cadre de cette initiative de recherche et fournit un aperçu de certaines des politiques et des recommandations sur les bonnes pratiques découlant de la mise en oeuvre des projets de recherche.
Conclusion: Les données produites par le projet de recherche devraient être prises en compte par les parties prenantes (y compris les collectivités, les décideurs, les praticiens de la santé publique et les autres partenaires concernés) afin de contribuer à une meilleure compréhension des répercussions des changements sociaux, environnementaux et climatiques sur les MTV (c'est-à-dire la nature du risque,

les vulnérabilités, l'exposition) et d’améliorer la capacité des pays africains à s’adapter à ces changements et à réduire les effets de ces derniers d'une manière qui soit bénéfique à leurs populations les plus vulnérables.

Translated from English version into French by Murielle Mettler, through

TRANSLATORS
WITHOUT BORDERS

Поддержка исследований, направленных на понимание уязвимых сторон системы здравоохранения в отношении трансмиссивных заболеваний: повышение устойчивости в условиях климатических изменений в Африке

Бернадетт Рамирез

Аннотация

Краткое описание. Заболевания, которыми люди заражаются от переносчиков болезнетворных организмов (ПБО), составляют 17 процентов всех инфекционных болезней и по-прежнему остаются значительной проблемой для общественного здравоохранения. В течение многих лет предпринимались большие усилия в борьбе с переносчиками болезней, в основном, за счёт внедрения крупномасштабных и скоординированных программ, которые способствовали снижению глобальной смертности, связанной с ПБО. Однако в связи с изменениями окружающей среды, включая изменение климата, ожидается, что воздействие на ПБО будет значительным с точки зрения связанных с ПБО опасностей, восприимчивости к инфекциям и подверженности риску воздействия. Несмотря на растущее осознание недостаточной защищённости африканского континента от ПБО в контексте изменения климата, в этой области все еще мало исследований и существуют препятствия для формулирования изменений в политике здравоохранения на основе фактических данных.

Основное содержание. Один из способов, с помощью которого можно восполнить пробел в знаниях и доказательствах, заключается в том, чтобы учреждения-доноры поддерживали исследования в этой области. Сотрудничество между Специальной программой ВОЗ по исследованиям и подготовке специалистов в области тропических болезней (TDR) и Международным центром исследований и разработок (IDRC) основано на более чем десятилетнем партнерстве в создании исследовательского потенциала в области тропических болезней. Благодаря этому партнерству появилась еще одна исследовательская инициатива по ПБО и последствиям изменения климата в Сахеле и Африке к югу от Сахары. В настоящем документе перечислены проекты, поддерживаемые в рамках этой исследовательской инициативы, и приводится краткая информация о некоторых рекомендациях в области политики и передовой практики, вытекающих из продолжающегося осуществления исследовательских проектов.

Вывод. Ожидается, что данные, полученные в результате исследовательской инициативы, будут использованы заинтересованными сторонами (включая общины, директивные органы, работников общественного здравоохранения и других соответствующих партнеров) для более

лучшего понимания воздействия социальных, экологических и климатических изменений на ПБО (т.е. характер опасности, восприимчивость к инфекциям, подверженность риску заболевания) и улучшить способность африканских стран адаптироваться и уменьшить последствия этих изменений способами, которые приносят пользу наиболее уязвимым группам населения этих стран.

Translated from English version into Russian by Natalia Potashnik, through

TRANSLATORS
WITHOUT BORDERS

Respaldo de la investigación conducida para comprender las vulnerabilidades de la salud de la población a enfermedades transmitidas por vectores: cómo aumentar la resistencia en condiciones de cambio climático en África.

Bernardita Ramírez

Resumen

Antecedentes: Las enfermedades transmitidas a las personas por vectores representan el 17 por ciento de todas las enfermedades infecciosas y continúan causando problemas importantes de salud pública. A través de los años, se ha logrado un gran progreso en la lucha contra enfermedades transmitidas por vectores (VBD, en inglés), en particular, mediante programas de control coordinados y a gran escala que han contribuido a la disminución de la mortalidad global atribuida a estas enfermedades. Sin embargo, debido a los cambios en el medio ambiente, incluidos climáticos, se prevé que el impacto en las enfermedades transmitidas por vestores serán significativos, en términos de los peligros, vulnerabilidades y exposición a estas enfermedades. Aunque hay una mayor concienciación sobre la vulnerabilidad del continente africano a las enfermedades transmitidas por vectores en el contexto del cambio climático, la investigación conducida en este ámbito es insuficiente, y la introducción de un cambio en las políticas, basado en evidencia, es imperiosa.

Sección principal: una de las formas en que se puede acortar la brecha del conocimiento y la evidencia es que las instituciones donantes apoyen la investigación en esta área. La colaboración entre el Programa Especial de la OMS para la Investigación y Capacitación en Enfermedades Tropicales (TDR, en inglés) y el Centro Internacional para la Investigación y el Desarrollo (International Center for Research and Development, IDRC) se basa en más de 10 años de cooperación en la formación de capacidades para la investigación, en el ámbito de las enfermedades tropicales. Otra iniciativa más de investigación de las enfermedades transmitidas por vectores y del impacto climático en el Sahel y el África Subsahariana surgió de esta asociación. Este documento enumera los proyectos respaldados por esta iniciativa de investigación y presenta un informe resumido sobre algunas de las recomendaciones de buenas prácticas y políticas, resultantes de la ejecución constante de los proyectos de investigación.

Conclusión: Se prevé que los datos generados de la iniciativa de investigación serán utilizados por grupos de interés (entre ellos, comunidades, legisladores, profesionales de salud pública y otros socios pertinentes), a modo de contribución, para que se comprendan mejor los impactos del cambio climático, ambiental y social en las enfermedades transmitidas por vectores (por ejemplo, la naturaleza del peligro,

las vulnerabilidades y la exposición), y se mejore la capacidad de los países africanos para adaptarse y reducir los efectos de estos cambios de forma tal que sea beneficiosa para sus poblaciones más vulnerables.

Translated from English version into Spanish by Sandra Bravo, through

TRANSLATORS
WITHOUT BORDERS
